# A Novel Flexible Model for the Extraction of Features from Brain Signals in the Time-Frequency Domain

**DOI:** 10.1155/2013/759421

**Published:** 2013-01-21

**Authors:** R. Heideklang, G. Ivanova

**Affiliations:** Institut für Informatik, Humboldt-Universität zu Berlin, Unter den Linden 6, 10099 Berlin, Germany

## Abstract

Electrophysiological signals such as the EEG, MEG, or LFPs have been extensively studied over the last decades, and elaborate signal processing algorithms have been developed for their analysis. Many of these methods are based on time-frequency decomposition to account for the signals' spectral properties while maintaining their temporal dynamics. However, the data typically exhibit intra- and interindividual variability. Existing algorithms often do not take into account this variability, for instance by using fixed frequency bands. This shortcoming has inspired us to develop a new robust and flexible method for time-frequency analysis and signal feature extraction using the novel *smooth natural Gaussian extension (snaGe)* model. The model is nonlinear, and its parameters are interpretable. We propose an algorithm to derive initial parameters based on dynamic programming for nonlinear fitting and describe an iterative refinement scheme to robustly fit high-order models. We further present distance functions to be able to compare different instances of our model. The method's functionality and robustness are demonstrated using simulated as well as real data. The *snaGe* model is a general tool allowing for a wide range of applications in biomedical data analysis.

## 1. Introduction

Electrophysiological brain signals are widely studied to get insights into the inner function of the brain. The electro-encephalogram (EEG), as an example, has been analyzed for decades and is particularly popular because of its noninvasiveness, wide availability, relatively small cost and excellent temporal resolution which enables capturing the fast neural dynamics. Because it is known that electrophysiological brain signals exhibit important spectral characteristics, frequency transforms are often applied. However, since in general brain signals do not possess the statistical property of stationarity, time-frequency transforms are of special interest. Such methods are able to represent a given signal jointly in the time-frequency domain, called its *time-frequency representation* (TFR). Thereby the signal's spectral components can be analyzed in relation to its temporal dynamics. In a wide sense, TFRs can be interpreted as images containing complex pixel intensities in general.

An important feature of biological signals, and particularly brain signals, is their inter- and intraindividual variability. That is, under fixed experimental conditions, the obtained signals exhibit heterogeneousness not only between groups of subjects, but also between subjects within the same experimental group and even within the same subject between multiple experimental trials. Existing signal analysis techniques either do not take this issue into account adequately or usually treat it by defining frequency bands of interest rather than single frequencies, and similarly time intervals instead of sharp instants. However, this strategy requires a priori knowledge about the variability to appropriately set the interval widths, and it is imprecise because it blindly includes all information contained in that time/frequency region.

A good example is the time-frequency coherence analysis of two given input signals, for instance, by means of the cross short-time Fourier transform [1], the cross Wigner-Ville distribution [2], or the wavelet coherence [1, 3]. All of these methods relate both signals at fixed time/frequency (or scale) locations. Thus, if signal A exhibits the same neural activation as signal B, but signal A's pattern is shifted in frequency just a little, none of the abovementioned techniques will be able to find the strong similarity of A and B. Although coherence estimation and other rigid strategies have been successfully applied “for more than 30 years” [4], this issue has inspired us to develop a general flexible method of *pattern analysis* and corresponding feature extraction in electrophysiological TFR data.

By abstracting from the TFR images and working with the representation of a TFR pattern, numerous applications in biomedical signal processing emerge. TFR patterns quantify neural activity and therefore extract useful features for subsequent analyses. The model proposed in this work goes even further by offering interpretable parameters. TFR patterns reduce dimensionality by representing neural activity in a wide spectrotemporal region by comparably few quantities. Pattern-based outlier detection has the potential to become a useful tool for data quality assurance. In ongoing studies we are employing the presented method, for instance, to estimate functional brain connectivity by means of a pattern-based approach.

In the following, we present the developed neuroinspired interpretable model which is able to capture general time-frequency patterns. We use solely EEG data for demonstrations here, but our method is applicable to general electrophysiological signals or even to other signals showing similar behavior.  Section 2 is devoted to developing our idea by extending the multivariate Gaussian model. Algorithms for robustly fitting the novel model to time-frequency representations are presented. A strategy for finding an appropriate model order is given, and distance functions are defined which quantify (dis-)similarity of two given models. These methods are tested in Section 3, where real as well as simulated data are used to demonstrate our technique's functionality and robustness.

## 2. Methods

While our technique is not restricted to specific time-frequency distributions, we employ the *smoothed pseudo Wigner-Ville distribution* [5] in this work. This is a quadratic transform estimating signal power in the time-frequency domain, whereby all quantities in this work are real numbers. The transform generates quite smooth TFRs, which means that neighboring pixel values are correlated. Although only positive values can be interpreted as signal power, the Wigner-Ville distribution introduces also negative values in general [6]. These data properties will be taken into account by our method.

We will refer to time-frequency representations as mappings *y* ^(TFR)^ : *T* × *F* → ℝ which estimate signal power for each point in the time-frequency domain.

Of the numerous ways to quantify TFR patterns, we choose to fit a parametric surface to the data. Because of the spatial correlation inherent in the data, traditional regression assumptions about independence of observations do not hold here [7]. This absence of strong gradients in the TFR images also invalidates most image feature extraction techniques, which are often based on edges and texture [8]. Our model, however, is especially designed for spatially correlated data; furthermore its parameters are interpretable. These quantities are useful features which embody important information about the underlying signal and thereby considerably reduce data dimensionality. Using our method, feature extraction can be fully automated, and no training data are necessary. Nevertheless, a priori information can be incorporated fairly easily.

In the following, we propose an extension of the well-known Gaussian model for TFR analysis.

### 2.1. The Gaussian Model

The Gaussian model for multivariate data *x* ∈ ℝ^*n*^ is defined by

(1) $$\begin{array}{c} y^{\left(G\right)}\left(x\right)=C+A\;\exp\left(-\frac{1}{2}{\left(x-\mu \right)}^{T}\Sigma^{-1}\left(x-\mu \right)\right) \end{array}$$

with *C* ∈ ℝ being a constant additive offset, *A* ∈ ℝ the amplitude relative to the offset, *μ* ∈ ℝ^*n*^ the constant *n*-dimensional mean vector, and Σ denoting a *n* × *n* symmetric positive definite matrix. Positive definiteness ensures that the argument to the exponential function is always negative; additionally we know that *e* ^*x*^ is bounded by zero and one for negative *x*. Therefore, the exponential factor scales the final amplitude between 0 and *A* relative to the offset *C*. The term (*x*−*μ*)^*T*^Σ^−1^(*x* − *μ*) is also known as the squared Mahalanobis distance of *x* with respect to *μ* and  Σ.

Because in our context this function represents arbitrary data in contrast to statistical distributions, *μ* will also be called the *position vector*, and Σ is the *spread matrix*, its entries *σ*_*ij*_ are denoted *spread parameters*.

Gaussian models are quite *robust* in various ways. Firstly, the model will be shaped like a peak for all possible parameter values by imposing the constraint that Σ (and thus also Σ^−1^) is symmetric positive definite. Thereby, the model will never be able to completely “degenerate.” Because the model is not flexible enough to fit small local variations of an expected pattern, the Gaussian model is relatively insensitive to local data outliers and is also unsusceptible to overfitting. An additional aspect of robustness is that extreme peak deformations are directly reflected in extreme parameter values. Thereby degenerated models can be easily detected or may even be prevented by imposing parameter constraints.

#### 2.1.1. Interpretability

The Gaussian model is well-suited to extract bivariate peaks from brain signals' TFR data, reflecting short intervals of neural excitement in a specific frequency range. An instance of the above described surface, in the bivariate case, is fully identified by its parameter vector

(2) $$\begin{array}{c} p={\left(C,A,\mu_{1},\mu_{2},\sigma_{11},\sigma_{12},\sigma_{22}\right)}^{T}. \end{array}$$

The absolute peak height *C* + *A*, the peak position (*μ*_1_, *μ*_2_)^*T*^, and the peak orientation can be derived from the parameter vector. Further relevant quantities are the temporal peak onset, peak offset, and peak duration (as the difference of the previous two).

### 2.2. Extending the Gaussian Model: The *snaGe* Model

As already mentioned, the Gaussian model's robustness comes at the cost of inflexibility. While some local effects in TFR data can be appropriately explained by (1), more general patterns of activation do not follow peak-like shapes, as will be shown later. Thus a generalization of the Gaussian model would be desirable, especially concerning the ability to represent *patterns of activation* rather than just independent “events” in the spectrotemporal domain. At the same time, a generalized method should maintain maximum robustness in order not to degenerate easily and to prevent overfitting. The so-called Gaussian mixture modeling (GMM) is a straightforward extension [9], but this method still assumes (multiple-) peak-shaped data. In the following, the smooth natural Gaussian extension (*snaGe*) model is presented as a flexible extension of the multivariate Gaussian model.

**Figure 1:**
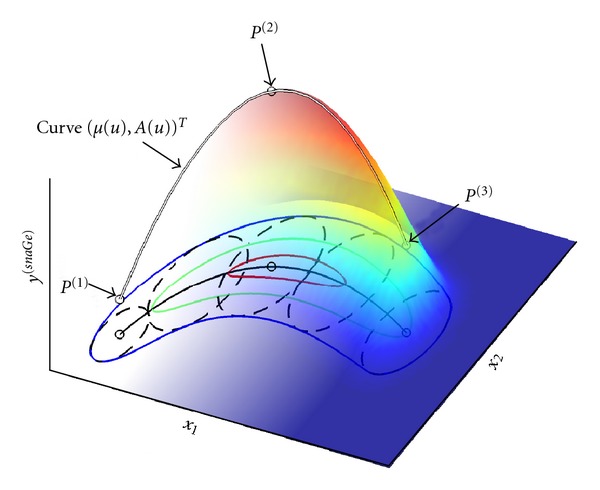
Example of an instance of the *snaGe* model, in the bivariate case. A surface plot is shown as well as its two-dimensional projection (colored contours). *K* = 3 three-dimensional points were smoothly interpolated to yield a “curve of peaks” (curve connecting the circles). This curve's 2d projection is *μ*(*u*), the black line. A surface is determined by the spread parameters (dashed 2d ellipses), controlling the shape of the exponential flattening to both sides of the curve.

Before giving a formal definition, we explain the idea in an intuitive way, guided by Figure 1. The *n*-variate Gaussian model can be described by its (*n* + 1)-dimensional peak point *P* = (*μ*_1_,…, *μ*_*n*_, *A*) and its spread parameters controlling the exponential flattening relative to *P* along the independent variables' dimensions. Now the idea is to not use only one, but *K* peak points *P* ^(*i*)^ ∈ ℝ^*n*+1^, *i* = 1 … *K*, interpolated by a smooth (*n* + 1)-dimensional curve of peaks. A way to think of the surface in Figure 1 modeled by *snaGe* is to “shape” it by sliding an *n*-variate Gaussian model along the curve, its peak point being connected to the curve and thus varying in height (*A*) and position (vector *μ*). Thereby complex smoothly “bent” patterns of data with varying amplitude (dependent variable) can be captured. The term *snaGe* is inspired by these snake-like forms. Analogously to the Gaussian model, an *n* × *n* spread matrix determines the model's shape, which is a surface for  *n* = 2.

The tradeoff between robustness and flexibility can be controlled by choosing the number of peak points *K*. Using many peak points will allow for good fits to complex patterns but will also increase the danger of overfitting. Choosing a small *K* yields a robust model, but its ability to capture complex patterns will be limited. By setting *K* = 1, *snaGe* reduces to the traditional Gaussian model as a special case.

Regarding the standard Gaussian model, each function value *y* ^(*G*)^(*x*) is fully determined by the Mahalanobis distance of the point *x* to the unique mean point *μ*. But since the *snaGe* model offers infinitely many mean points, the question arises how to calculate the surface values. This issue will be addressed in the next section, where a formal definition is given.

#### 2.2.1. Formal Definition

Let the “number of peak points” be denoted by *K* ∈ *ℕ*, *K* ≥ 1. Let *μ* : [1, *K*]→ℝ^*n*^ denote a smooth *n*-dimensional curve of “means,” and let *A* : [1, *K*]→ℝ be a smooth one-dimensional curve of “amplitudes” along *μ*. Let further the “offset” *C* ∈ ℝ, and let Σ ∈ ℝ^*n*×*n*^ be a symmetric positive definite matrix. Define

(3) y~(snaGe):ℝn×[1,K]→ℝy~(snaGe)(x,u)≔C+A(u)  exp(−12(x−μ(u))T         ×Σ−1(x−μ(u))).

Note that ${\tilde{y}}^{\left(snaGe\right)}$ is a family of traditional Gaussian models, parameterized by the curve parameter *u*. In order to construct a function which is independent of *u*, we define

(4) $$\begin{array}{c} u^{*}\left(x\right)≔\arg\underset{u\in \left[1,K\right]}{\max}\left|{\tilde{y}}^{\left(snaGe\right)}\left(x,u\right)-C\right|. \end{array}$$

The *snaGe* model is then given by

(5) $$\begin{array}{c} y^{\left(snaGe\right)}:{\mathbb{R}}^{n}\to \mathbb{R}\\ y^{\left(snaGe\right)}\left(x\right)≔{\tilde{y}}^{\left(snaGe\right)}\left(x,u^{*}\left(x\right)\right). \end{array}$$

The function u*(*x*) defines that traditional Gaussian model which assigns to *x* the largest absolute amplitude relative to the offset *C* among all members of the Gaussian family ${\tilde{y}}^{\left(snaGe\right)}$. This is necessary to cope with positive as well as negative *A*(*u*). In TFR analysis, *A*(*u*) can be restricted to only positive values (see Section  2.3.1), in which case (5) in fact simplifies to

(6) $$\begin{array}{c} y^{(snaGe)+}\left(x\right)≔\underset{u\in [1,K]}{\max}{\tilde{y}}^{\left(snaGe\right)}\left(x,u\right). \end{array}$$

**Figure 2:**
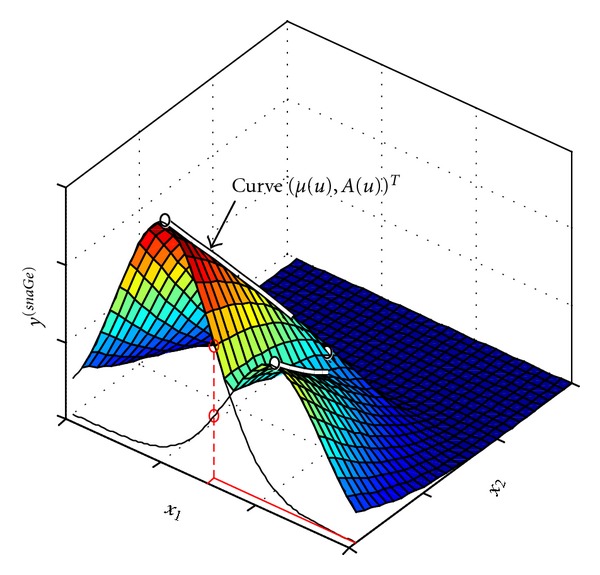
Depiction of the maximum rule. The curve of means *μ*(*u*) is sharply bent. The surface value for a point (*x*_1_*, *x*_2_*) (coordinates depicted by red lines) is chosen among all possible traditional Gaussians (black graphs visualize 1D cuts of two of these) by the maximum rule (red circles), see (6).

For *y* ^(*snaGe*)^ as well as *y* ^(*snaGe*)+^ the max( ) function is necessary in case *μ*(*u*) is a “near self-intersecting” curve, in the sense that ||*μ*(*u*_1_) − *μ*(*u*_2_)|| is small, but |*A*(*u*_1_) − *A*(*u*_2_)| is large.  Figure 2 illustrates such an exemplary scenario.

We assume that the diagonal entries of the spread matrix Σ, that is, the spread along each dimension, are sufficient to control a TFR pattern's “width.” Therefore, we fix offdiagonal entries to zero for the sake of robustness. Thereby, the Mahalanobis distance in (3) reduces to the weighted Euclidean distance.

The two curves *A*(*u*) and *μ*(*u*) are yet to be defined in terms of discrete parameter values. For good parameter interpretability, we choose to form both curves by interpolating *K* points *P* ^(*i*)^ ∈ ℝ^*n*+1^, *i* = 1 ⋯ *K* by B-splines of degree ≤3, which yields the curve:

(7) P:[1,K]→ℝn+1,  P(u)=(P1(u)…Pn(u)Pn+1(u))=(μ1(u)…μn(u)A(u))=(μ(u)A(u)).

By combining cubic B-splines with the Gaussian shape, we obtain a sufficiently smooth model which inherits both the splines' flexibility and the Gaussian standard model's robustness. Our model further inherits the B-splines' *local control* property; that is, varying a *P* ^(*i*)^ will affect the model only in the *P* ^(*i*)^'s vicinity. Additionally, the degree of flexibility can be adapted to the data at hand by varying *K* from 1 (single Gaussian peak) to arbitrary flexibility with *K* > 1. 

An instance of the *n*-variate *snaGe* model with *K* points (or *of order K*) is fully represented by the parameter vector of length 1 + *n* + (*n* + 1)*K*:

(8) $$\begin{array}{c} p={\left(C,\sigma_{11},\sigma_{22},\ldots ,\sigma_{nn},P_{1}^{\left(1\right)},\ldots ,P_{n+1}^{\left(1\right)},\ldots ,P_{1}^{\left(K\right)},\ldots ,P_{n+1}^{\left(K\right)}\right)}^{T}. \end{array}$$

While the offset *C* and the spread parameters are mainly responsible for the prediction of data values, the curve *P*(*u*) interpolating the *P* ^(*i*)^ is directly interpretable as it models the main *path of peaks* in the data.

In the next section we show how to fit the model to data in a robust manner.

### 2.3. Fitting the Model to TFR Data

Given a time-frequency representation *y* ^TFR^(*x*), *x* ∈ *T* × *F*, we aim to find a parameter vector *p* so that the respective model *y* ^(*snaGe*)^ fits the data “best,” in the sense that it minimizes a cost function. We use the sum of squared differences of the data and the modeled surface:

(9) SSE(p)=∑x=(t,f)Tt∈T,f∈F(y(TFR)(x)−y(snaGe)(x))2.

The parameters *p* are implicitly represented by *y* ^(*snaGe*)^ in the above formula. This quantity is also called the “sum of squares due to error” which is zero if the model perfectly fits the data. SSE is a nonlinear function dependent on *p*. Using squared differences pronounces outliers, but these are not expected to occur frequently in our smooth TFR data. In order to find a locally minimum solution of SSE, a nonlinear least squares algorithm implemented in the MATLAB Optimization Toolbox, *lsqnonlin* [10], is employed. Given an initial parameter vector and the cost function SSE, this optimizer produces a sequence of models *p*_*i*_. The iteration hopefully converges to a p* with minimum cost, that is, best resemblance between model and data.

We attempt to provide advantageous starting conditions for the optimizer by preprocessing the TFR and by obtaining an initial parameter vector *p*_0_ which is expected to be close to the optimum with respect to SSE. Moreover, an iterative refinement scheme is proposed to be able to robustly fit models of high order. The process of fitting is outlined by Figure 4.

#### 2.3.1. TFR Preprocessing

TFR data are badly scaled, showing differences of several orders of magnitude in values of time, frequency, and signal power, which affects optimization performance [11]. To address this problem, *lsqnonlin* offers a way to take into account typical values for each dimension for gradient estimation. Also concerning this issue, any Euclidean distance operating on TFR data in our algorithms is weighted appropriately. Furthermore, smooth objective functions are desirable so that the low-order Taylor approximations used during optimization resemble the cost function in a relatively large neighborhood around the current point. To this end, the TFR images are smoothed and subsampled, which has the additional benefit of faster cost function evaluations. Finally, since negative values in the time-frequency domain are not interpretable, they are usually set to zero.

#### 2.3.2. Estimation of Initial Parameters

Generally, nonlinear cost functions may exhibit multiple local extreme values. Since *lsqnonlin* performs local optimization, a start parameter vector should be chosen such that it already lies in the basin of the cost function's (unknown) global minimum, that is, a vector whose cost is already low. To this end, the constant offset and the spread parameters are initialized to *C* = 0, *σ*_11_ = (*t*_max_ − *t*_min_)/5 and *σ*_22_ = (*f*_max_ − *f*_min_)/5 if the underlying TFR's time and frequency axes are bounded by *t*_min_, *t*_max_ and *f*_min_, *f*_max_, respectively. One may choose any sensible values alike, but the spreads should not be initialized too small in order to obtain a generalizable model. Yet, more thought has to be put in choosing the number and coordinates of the peak points *P* ^(*i*)^. In the following we propose a way to compute initial estimates of the time, and frequency coordinates of all *P* ^(*i*)^ directly from the data.

**Figure 3:**
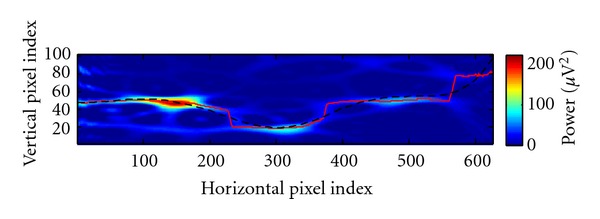
Illustration of an optimal horizontal image path (red line) found by a dynamic programming algorithm. The sum of signal power values along the red line is higher or equal to that of any other horizontal path. The trajectory is smoothed (black) for the robust extraction of the initial peak points' time-frequency coordinates. The background TFR image is computed from simulated data, whose three consecutive peaks of activity are correctly connected by the path.

**Figure 4:**
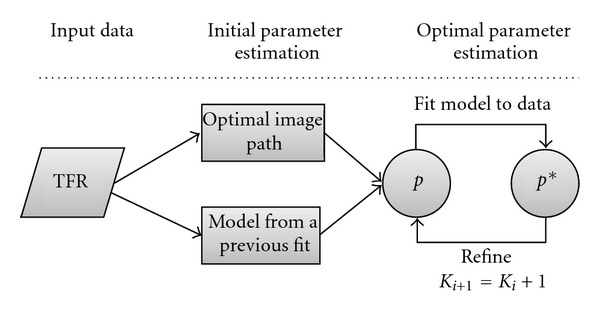
Flow chart of the fitting process. Initial parameters are derived from the TFR data, or a previously fitted model is used to determine *p*. The model is fitted using a nonlinear optimization algorithm. If desired, the optimal model can be iteratively refined up to *K*_max_.

A reasonable approximation to the unknown optimal curve of peaks can be found by tracing a path through the TFR image *y* ^(TFR img)^ from left to right, which runs through areas of high pixel intensity. More precisely, the sum of all pixel intensities along this path should be as high as possible. This is an optimization problem in turn, yet its solution can be computed in quadratic time complexity (provided that the path's slope is bounded) by a dynamic programming algorithm, see [12]. Because a global optimum is guaranteed to be found, this strategy is insensitive to local outliers and noise. To this end, a similar approach as described in [13] is employed. Following the notation therein, we define our energy function to be equal to the TFR values themselves, that is, *e*(*I*) = *I*. A horizontal path s* (called *seam *in [13]) which maximizes (this is in contrast to [13], where *minimum* energy seams are computed) this simple cost function is found by dynamic programming. See Figure 3 for an example. Additionally, the paths are constrained by imposing an upper bound *k* on their slopes. This value depends on the time-frequency resolution here and once more represents a compromise between robustness and flexibility.

Given the found path s* and the desired model order, *K* evenly spaced samples are subsequently drawn from a smooth approximating curve to obtain estimates of the first two coordinates of the *P* ^(*i*)^, *i* = 1 ⋯ *K*. We choose to empirically set the *P* ^(*i*)^s' last components, interpretable as amplitudes relative to the initial constant offset *C* = 0, to max(*y* ^(TFR)^) − *C* = max(*y* ^(TFR)^).

Once a parametric representation of the data is available, its accuracy can be improved in a step-wise manner, as is presented in the following section.

#### 2.3.3. Iterative Refinement

As already stated, the number of points *P* ^(*i*)^ controls the model's robustness which complements its ability to resemble complex patterns. Therefore, the demand for a near-optimal initial parameter vector increases with the model order *K*. Employing the optimal image path method described in the previous section yields a “reasonable” estimation, but sampling *K* equidistant points *P* ^(*i*)^,   *i* = 1 … *K* from the resulting curve is a simplification. In fact, it can be observed that the optimizer tends to concentrate the *P* ^(*i*)^ in time-frequency regions of high signal variability. For low model order *K*, this shortcoming of our initial parameter estimation algorithm can be compensated easily by the optimization algorithm, but it may become a problem for increasingly flexible models. For this reason we propose an iterative scheme.

1. Find initial parameters *p*_0_ by means of an optimal path (see Section  2.3.2) for a first, robust model of low order *K*_0_. Let *i*≔0.
2. Fit the model to the data to obtain optimal parameters *p*_i_*.
3. Construct the optimal curve of peaks *P*(*u*) by interpolation of the peak points (see Section  2.2.1).
4. Obtain the *K*_*i*+1_ = *K*_*i*_ + 1 peak points for a refined model by uniformly sampling (with respect to the spline's sites *u*_*j*_) the curve computed in the previous step.
5. Construct the parameter vector *p*_*i*+1_ of the refined model from *p*_*i*_ by replacing the old *K*_*i*_ peak points with the *K*_*i*+1_ new ones.
6. If *K*_*i*_ < *K*_max_, let *i*≔*i* + 1 and continue with step 2.

The curve found in steps 2 and 3 will exhibit smaller gradient magnitude, that is, traversal speed, in areas of high signal variability than in other regions. We aim at maintaining the curve-defining points' optimum distribution found by the fitting algorithm and at enhancing the model's flexibility mainly in these areas. It turns out that simply by uniformly sampling the fitted curve (step 4) we obtain a new interpolated curve which retains these properties.

An application of this algorithm is demonstrated in Section  3.1, where the resulting sequence of nested models is evaluated.

### 2.4. Model Distance

In this section we propose two functions for calculating the distance between two models *y*_1_^(*snaGe*)^, *y*_2_^(*snaGe*)^ regarding (dis-)similarity of shape. Distance measures are necessary, for instance, to quantify how well the data exhibit an expected pattern. We will also employ these functions to assess our model's robustness.

Distance functions which are based solely on the curve of peaks *P*(*u*) were found to be quite effective. Other possibilities include parameter vector distances and pixel-wise differences of signal power of the models' generated data. By comparison, curve-based distance functions have the advantage of being able to interrelate models of different orders. Additionally, they are not influenced by the less informative parameters (offset *C* and the entries of Σ).

A popular distance measure for parametric curves is the *Fréchet distance* [14]. In the continuous case, the Fréchet distance of two parametric curves *P*_1_(*u*) and *P*_2_(*u*) is defined by

(10) $$\begin{array}{c} D_{F\_\max}\left(P_{1},P_{2}\right)=\underset{\alpha ,\beta }{\inf}\;\underset{u}{\max}\;d\;\left(P_{1}\left(\alpha \left(u\right)\right),P_{2}\left(\beta \left(u\right)\right)\right). \end{array}$$

Here, *α*(*t*) and *β*(*t*) are monotone reparameterizations of the two curves, and *d*( ) denotes (weighted) Euclidean distance. In words, we search for those reparameterizations which make the curves the most similar with respect to maximum point-wise Euclidean distance along the curves. This maximum for these reparameterizations is returned as the two curves' continuous Fréchet distance. In practice, the *discrete* Fréchet distance is frequently applied, whose computation is based on dynamic programming once more [15]. In the discrete case, an additional distance function *D*_*F*_sum_(*P*_1_, *P*_2_) can be obtained by replacing the max function with a *sum* over *u*. That way, *D*_*F*_sum_ represents an average distance, being less prone to outliers in the curves.

## 3. Results

### 3.1. Real Data

**Figure 5:**
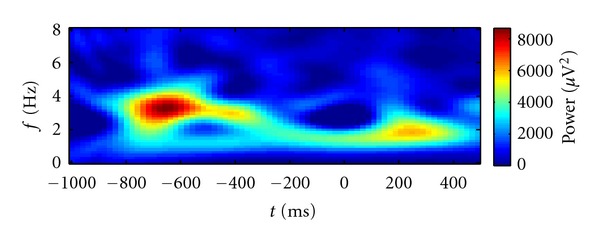
TFR of real EEG data which is fitted by iteratively refined models. Multiple local peaks are visible which are connected by a path of increased activity. This forms a complex pattern.

We demonstrate the workflow to determine the appropriate model order *K* by fitting a TFR of real EEG data in this section. Typically we determine the necessary model complexity by fitting data with good signal to noise ratio (SNR) in order to prevent the overestimation of *K*. For example, one possibility to achieve sufficient data quality is to average several TFRs which are expected to show similar patterns. The averaged TFR of real EEG data shown in Figure 5 will guide the following explanations. The depicted brain signals located in the lower frequency bands were recorded from the temporal brain region during a face recognition experiment. These data exhibit a pattern of activity which is too complex to be captured by a traditional Gaussian model.

The iterative scheme described in Section  2.3.3 is employed to fit models of increasing flexibility to the high-quality data. An optimal path (see Section  2.3.2) estimates the initial parameters for the first, least flexible model of order *K*_0_. A minimum value of *K*_0_ = 3 is necessary to model bent patterns. Since the appropriate *K* is still unknown, a sufficiently large number *K*_max_ = 7 is chosen for the refinement. At each refinement stage the respective model is evaluated, and in the end the most suitable

(11) $$\begin{array}{c} K^{*}\in \left\{K_{0},\ldots ,K_{\max}\right\},\;K_{0}\leq K^{*}\leq K_{\max} \end{array}$$

is chosen as the model order for future fittings on lower-quality data. Model evaluation is realized by three measures. These are the cost function value (SSE, see Section  2.3), the coefficient of determination *R* ^2^ and its adjusted version *R*_adj_^2^ [16]. Although the use of quantities based on the coefficient of determination is discouraged for nonlinear models [17], they are applied here nonetheless for two reasons. They are found to perform well for our purposes, and the proposed alternatives (AIC [18] and BIC [19]) are not easily applicable here. This is because the assumption of normally distributed residuals often does not hold, which is supported by a highly significant Shapiro-Wilk test [20] at *α* = 5% yielding *p* < 10^−3^ for this experiment.

**Figure 6:**
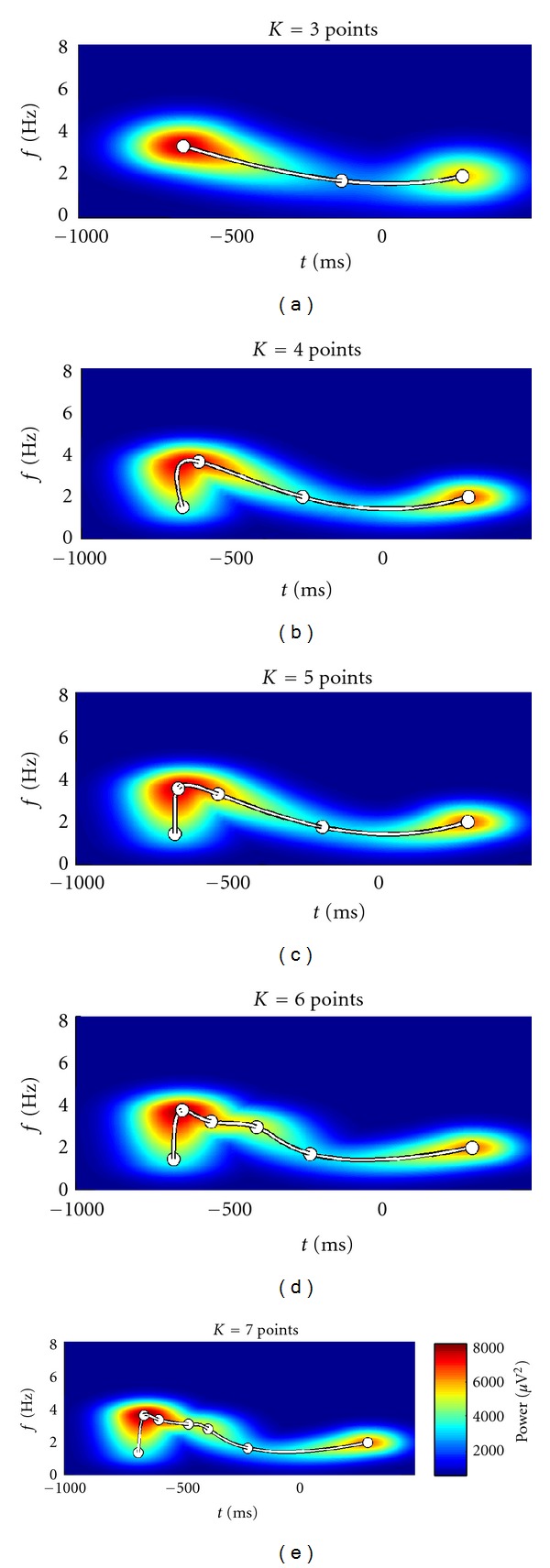
Surfaces of 5 iteratively refined models. Increasing the model order *K* clearly enhances the models' flexibility.

**Figure 7:**
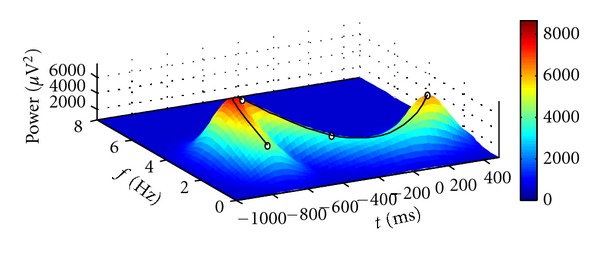
Three-dimensional plot of the fitted model of order *K* = 4. The black line represents the curve of peaks.

**Figure 8:**
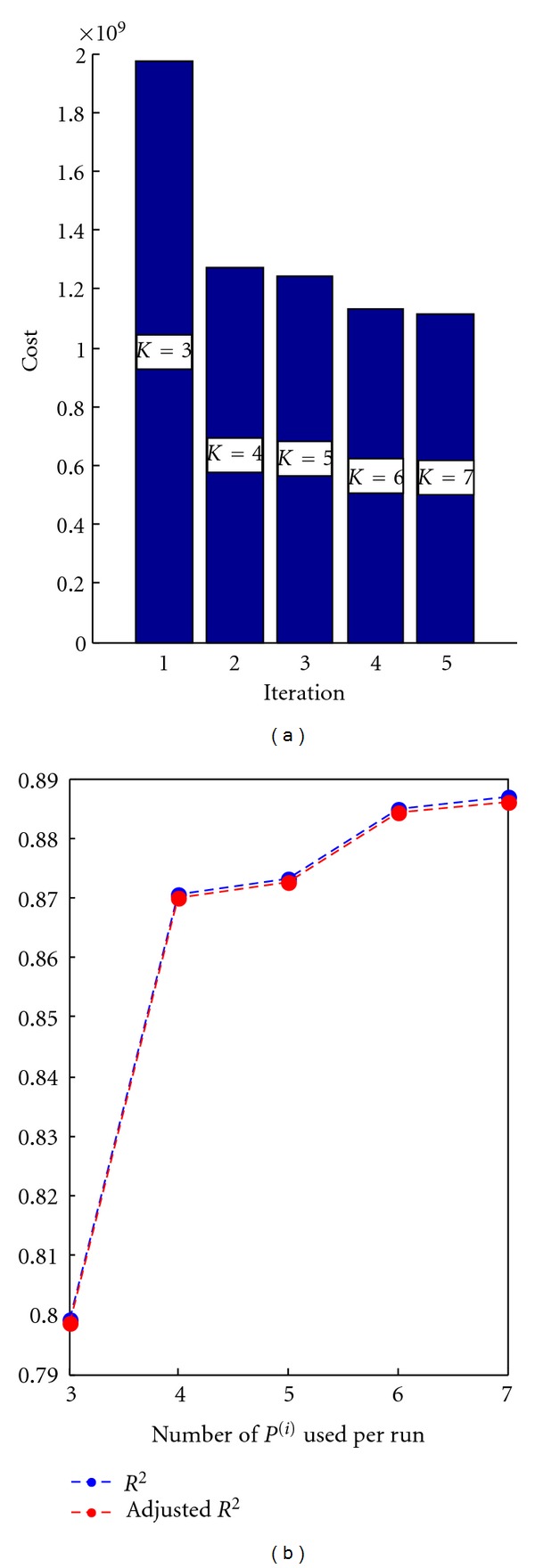
Goodness of fit for each iteration. (a) Cost function value (SSE). (b) Coefficient of determination *R* ^2^ and adjusted *R* ^2^. Apparently, the data require the flexibility of a model with at least *K* = 4. Adding further points does not improve the model as much anymore and therefore K* = 4 is a reasonable choice.

Figures 6, 7, and 8 illustrate the results.

The results show that for the data at hand a model of order K* = 4 is sufficient to capture the variability.

Having determined the maximum model complexity on high-quality data, such a model can now be fitted to the rest of the data. If the TFRs are not expected to vary substantially, like when fitting a model to signals from several nearby sensors, a previous fit may serve as the initial model. However, if, for instance, multiple data segments of the same sensor should be fitted, the TFRs' patterns may vary strongly. In this case, initial parameters should be chosen depending on the data by using the method of optimal paths described in Section  2.3.2. Since in this example K* = 4 is a quite moderate number, the iterative refinement may also be skipped. However, in general we would start with *K*_0_ = 3 or *K*_0_ = 4 and refine up to the determined K*, as proposed in Section  2.3.3.

### 3.2. Synthetic Data

We want to assess our model's robustness by simulating data and measuring how strongly the model is affected by additive Gaussian noise. To this end, artificial data are created in the time domain, and their TFRs are computed to which our model will be fitted. 

#### 3.2.1. Description of the Simulated Data

**Figure 9:**
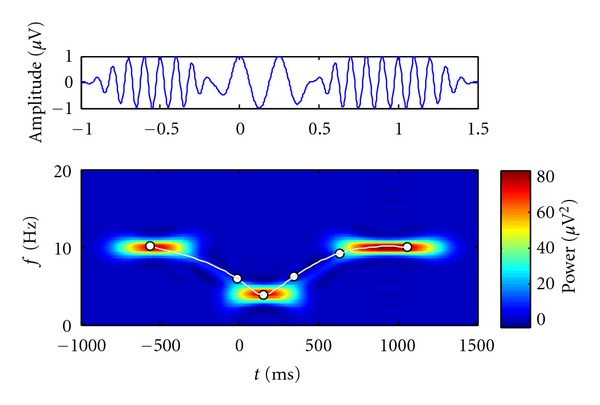
The simulated signal in the time domain and in the time-frequency domain showing a complex alpha/theta/alpha oscillation pattern. The white line with dots represents a fitted model which will serve as the reference model for the experiments.

We created a signal consisting of three consecutive oscillations, representing an alpha-theta-alpha EEG pattern at 10 Hz/4 Hz/10 Hz respectively over a time span of 2.5 seconds. The simulated sample rate is 250 Hz. A plot is shown in Figure 9. These data are quite challenging for our model because three distinct peaks emerge in the TFR which could be more appropriately modeled by a mixture of independent Gaussian peaks. However, we want to demonstrate the flexibility of the *snaGe* model which should also be able to cope with patterns of this form.

For the following experiments we chose to start the fitting with a model of order *K* = 5 to account for the pattern's complexity and perform one refinement step. Initial parameters are estimated by finding optimal paths, which means that no a-priori information about the known optimal model is passed to the fitting procedure other than the number of *P* ^(*i*)^ to use. We define the optimal model by fitting the noise-free simulation in the same way. The distance measures from Section  2.4 are used to determine how well the simulated pattern is found.

#### 3.2.2. Noise Experiment

In this experiment we added Gaussian noise, which is appropriately filtered with respect to the sampling frequency, to the simulated data in the time domain. Signals exhibiting signal to noise ratios of −15 dB up to +10 dB were generated in steps of 2.5 dB. At each SNR, ten distinct noise realizations are created to obtain representative results. This independent noise in the time domain will produce correlated noise in the time-frequency domain due to smoothing. Therefore, the pattern shown in Figure 9 will be distorted. This experiment serves to assess how strongly our algorithm is affected by pattern variability, respectively, to investigate its robustness. Small pattern distortions should ideally only slightly alter the optimal model, reflecting its robustness and avoidance of overfitting. We further want to find out to what degree our model is able to find the simulated pattern at all.

We note here that adding noise increases the TFRs' maximum amplitudes exponentially which strongly affects the comparability of different models. Without normalization, one would observe an exponentially decreasing distance for increasing signal to noise ratio. But this would merely reflect the decreasing data amplitudes and contain no information about the quality of fit. However, normalizing maximum data values are not an appropriate option either, because, for negative SNRs, this would keep the noise constant while exponentially shrinking the pattern's pixel intensities. Even if the optimal model was perfectly recovered from the noisy simulation, high distances would arise. Only if signal power is excluded from model distance estimation, the returned values are useful representatives of how well the pattern was found. The *snaGe*'s robustness to noise with respect to the pattern's power is therefore not regarded here. This is done by setting the third dimension of the path of peaks *P*(*u*) to zero during Fréchet distance computation.

**Figure 10:**
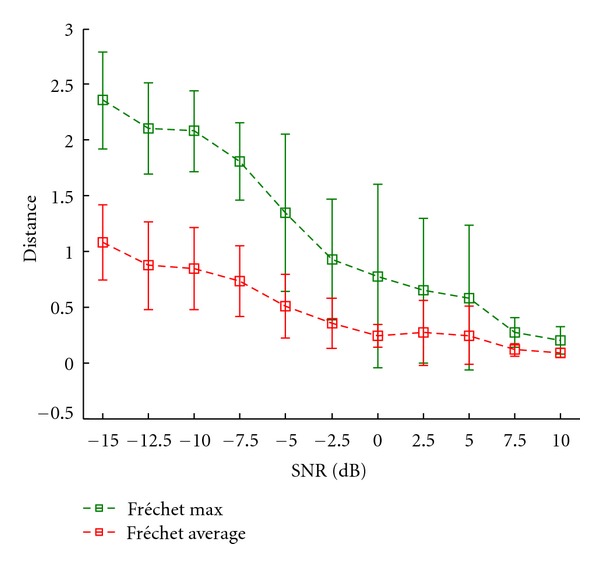
Mean distance to the optimal model versus level of correlated noise in the time domain. Error bars represent one standard deviation.

**Figure 11:**
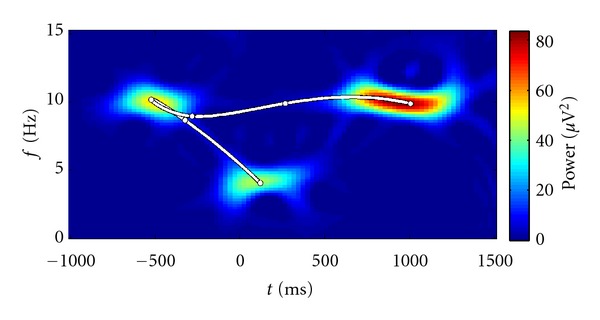
Fitted model (white curve) at SNR = 5 dB, which has the largest distance *D*_*F*_sum_ to the target pattern across all ten noise instances. The noisy TFR is shown in the background. The signal was successfully extracted, yet a high-distance results due to a different connection of the three peaks compared to the pattern.

Figure 10 visualizes the results. Both curves of mean distance consistently decrease with improving data quality. Convergence to the optimal model seems to require high signal to noise ratios. At 7.5 dB, the distance measures' variances fall off, reflecting the point of reliable pattern extraction. Apparently, the noise and interferences introduced in this experiment considerably impair the fitting process. In Figure 11, this issue is exemplarily investigated. At the positive SNR of 5 dB, where distance variances across the noise realizations are still high, the fit which exhibits the largest distance is plotted. The pattern was in fact found, but only in a different way than was expected. This leads to high Fréchet distances. Nevertheless, this example shows that the impact different kinds of noise may have on the fitting process.

**Figure 12:**
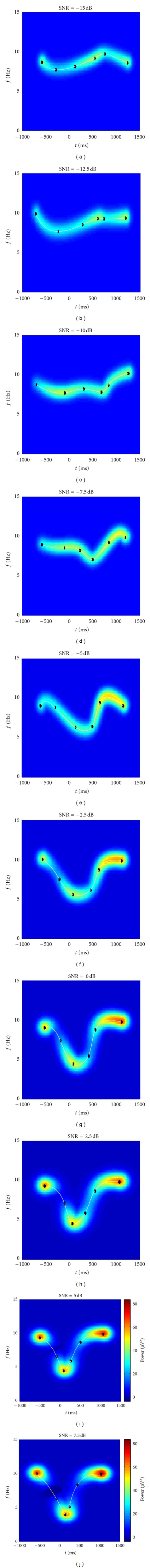
Mean *snaGe* models per simulated SNR. Models were averaged across the ten noise realizations by direct parameter vector averaging. The curve of peaks (white line) is shown as well as the models' predicted data *y* ^(*snaGe*)^(*t*, *f*). Compare with Figure 9.

To get a better feel for the average ability to fit the pattern under the influence of noise, see Figure 12. At each noise level, the ten fitted models are averaged by computing the mean parameter vector. Shown is a sequence of mean models which progressively look more similar to the true pattern. In fact, the average fitting capability concerning both the positioning of the peak points in the time-frequency domain and the estimation of surface values is better than expected after having studied Figure 10. Apparently, although the mean distances are still decreasing at negative signal to noise ratios, they are already small enough for successful pattern extraction on average. An example is the subplot corresponding to SNR = 0 dB in Figure 12, which already clearly resembles the simulated pattern.This experiment shows that interferences between the desired signal and additive noise affect the fitting process quite strongly in the worst case. Positive signal to noise ratios of at least 7.5 dB are found to be necessary for reliable pattern extraction in this investigation. However, successful data modeling is also possible at lower SNRs, as is seen in the average case.

## 4. Discussion

The *snaGe* model is especially suited for time-frequency representations of electrophysiological signals because of their (expected) nonnegativity, smoothness and their patterns following a path of peaks. However, our robust model is able to cope with data which do not exactly meet these requirements.

In order to retain robustness, we imposed several restrictions on our model, like neglecting offdiagonal spread parameters and holding the spread matrix constant over the curve of peaks. An interesting question remains how the model's flexibility and robustness would be affected if these constraints were dropped. Little effort would be necessary to include the stated extensions.

As is typical for nonlinear optimization problems, the choice of initial parameters is crucial to obtain satisfying results. Therefore, a-priori knowledge about the optimal model can be incorporated by starting the optimization with a model which was previously fitted to similar data. Moreover, an algorithm based on an optimal path is developed to estimate initial model parameters directly from the data. Its robustness stems from the guarantee to find the globally optimal path. However, this method is limited to *positive* peak polarity by trying to *maximize* the path's average amplitude. Additionally, the technique will not be able to find initial models which exhibit multiple contemporary components. In such a case, the nonlinear optimization algorithm, which was found to work well, must compensate. Further strategies for the estimation of initial parameters would be desirable. In particular, the extraction of the curve interpolation points *P* ^(*i*)^ from the optimal path possesses potential for improvement.

An open question is how we should deal with the spatial correlation of both the dependent variable and the residuals in a statistical inferential context. Further work is necessary to facilitate statistical testing, for instance, to assess the null hypothesis that an expected pattern is not contained in the data.

Concerning the presented measures of model distance, the Fréchet distances were found to be very useful to assess model similarity in our experiments involving simulated noise. Their distinct advantage is their independence of model order and the disregard of the less interpretable parameters. On the other hand, spurious high distances could be observed when in fact the pattern was found. This can be attributed to the fact that the simulated data exhibit three independent peaks, which is a violation of the *snaGe*'s assumption of a connected path of peaks. Therefore, a combination of Fréchet values and a pixel-wise distance function based on the models' generated data seems advantageous.

When applied to noisy time signals, the *snaGe* model adapts too well to the corresponding smooth time-frequency representations. Because the optimized cost function does not take into account information about the expected pattern, the model simply tries to capture the TFR data as accurately as possible. Data preprocessing and TFR interference suppression are therefore extremely important. Adding penalty terms to the cost function and/or providing explicit initial parameters are ways to point the optimizer in the right direction. However, even without specifying a-priori knowledge, the model was able to find the simulated pattern for low signal to noise ratios in the average case.

## 5. Conclusion

The analysis of time-frequency representations of electrophysiological signals calls for flexible methods accounting for inter- and intraindividual data variability. We present the flexible, robust, and interpretable model *snaGe*, which extends the established Gaussian model. Its ability to extract 3D features from time-frequency representations of electrophysiological data is demonstrated. However, the model applies to general multivariate data which exhibit similar behavior.

In this work, several techniques to improve the model fitting performance are described. We show how to estimate start parameters directly from the data. An iterative scheme to refine optimized models is proposed so that high-order models can be robustly fitted.

Experiments with real as well as simulated data demonstrate the *snaGe* model's robustness and flexibility. Under the influence of severe noise, the developed technique is best suited for patterns which are too complex to be appropriately captured by a Gaussian model, but still simple enough to facilitate robust fits.

To summarize, due to its robustness and flexibility the *snaGe* model possesses the potential to become a beneficial tool for practical EEG/MEG analysis, including functional brain connectivity analysis, outlier detection, time-frequency denoising, and feature extraction.
